# Macrophages in tissue repair and regeneration: insights from zebrafish

**DOI:** 10.1186/s13619-024-00195-w

**Published:** 2024-06-11

**Authors:** Changlong Zhao, Zhiyong Yang, Yunbo Li, Zilong Wen

**Affiliations:** 1 Department of Immunology and Microbiology, School of Life Sciences, Southern University of Science and Technology, Shenzhen, 518055 China; 2 Division of Life Science, the Hong Kong University of Science and Technology, Clear Water Bay, Hong Kong, China; 3 Shenzhen Bay Laboratory, Shenzhen, 518055 China

**Keywords:** Macrophage, Repair and regeneration, Ontogeny, Heterogeneity, Plasticity, Inflammation, Tissue remodeling, Zebrafish

## Abstract

Macrophages play crucial and versatile roles in regulating tissue repair and regeneration upon injury. However, due to their complex compositional heterogeneity and functional plasticity, deciphering the nature of different macrophage subpopulations and unraveling their dynamics and precise roles during the repair process have been challenging. With its distinct advantages, zebrafish (Danio rerio) has emerged as an invaluable model for studying macrophage development and functions, especially in tissue repair and regeneration, providing valuable insights into our understanding of macrophage biology in health and diseases. In this review, we present the current knowledge and challenges associated with the role of macrophages in tissue repair and regeneration, highlighting the significant contributions made by zebrafish studies. We discuss the unique advantages of the zebrafish model, including its genetic tools, imaging techniques, and regenerative capacities, which have greatly facilitated the investigation of macrophages in these processes. Additionally, we outline the potential of zebrafish research in addressing the remaining challenges and advancing our understanding of the intricate interplay between macrophages and tissue repair and regeneration.

## Background

Tissue repair and organ regeneration are crucial processes for maintaining the structural integrity and functionality of organisms following injury. These intricately organized processes rely on effective collaboration between tissue and immune cells. Among the immune cells, macrophages are central in orchestrating various aspects of the repair process, including initiating and resolving inflammation, clearance of debris and foreign agents, and tissue remodeling (Wynn and Vannella 2016). Their pivotal involvement has also sparked significant interest in exploring macrophages as potential therapeutic targets for diseases associated with impaired tissue repair and regeneration (Aurora and Olson 2014). However, the precise mechanisms by which macrophages execute and coordinate these diverse functions during the repair process remain undefined, hindering the development of effective therapies.

For a long time, macrophages’ multifaceted features and functions were attributed to their exceptional plasticity. It was believed that macrophages could dynamically alter their molecular and cellular profile in response to injury and repair signals, through which they acquire specific attributes and perform proper functions at different stages of the repair process (Kim and Nair 2019). However, recent studies have revealed that macrophages in most organs are heterogeneous populations comprised of ontogenically and functionally distinct subsets (Ginhoux and Guilliams 2016). It is thus suggested that these macrophage subsets may fulfill different functional aspects during the repair process (Wynn and Vannella 2016). Currently, there is growing acceptance that both compositional heterogeneity and functional plasticity of macrophages contribute to their intricate regulatory functions during tissue repair and regeneration. Therefore, identifying the distinct macrophage subpopulations and unraveling their dynamic roles in the repair process has been the central focus of research endeavors. However, due to the inherent technical complexities involved, the precise identification of distinct macrophage subpopulations and the analysis of their specific responses and functions during the repair process in mammalian studies has been challenging (Mihlan et al. 2022).

Owing to its remarkable regenerative abilities, zebrafish has emerged as a prominent model organism for investigating tissue repair and regeneration. Its genetic tractability and the readiness of intravital imaging enable in vivo molecular and cellular characterization of cells involved in the repair process in unprecedented detail. Extensive studies in zebrafish over the past decade have provided valuable insights into understanding the development and functions of macrophages in both homeostasis and repair conditions (Bohaud et al. 2021; Keightley et al. 2014). Many of these findings have demonstrated conservation across higher organisms, underscoring their broader relevance. In this review, we summarize the current understanding of macrophage biology in tissue repair and regeneration, with a particular focus on the contributions made by zebrafish studies.

## Zebrafish as a prominent model for macrophage and regeneration study

Zebrafish was first introduced to the scientific community by George Streisinger as a model organism for studying vertebrate development and genetics. Recently, it has gained growing recognition for its distinct advantages in investigating immunobiology. As a vertebrate, zebrafish possess a conserved immune system comprising innate and adaptive immune cells (Bjorgen and Koppang 2021). Besides, zebrafish share approximately 70% genetic homology with humans (Howe et al. 2013). Therefore, the molecular understanding of immunity obtained from zebrafish studies can have broader implications for higher organisms. Moreover, the large clutch size and low maintenance cost of zebrafish make it ideal for high-throughput drug and genetic screening, facilitating the identification of potential therapeutic targets or drug candidates for immune disorders and diseases.

Unlike mammals, zebrafish undergo external embryonic development and possess a transparent body during the early stages of development. This unique feature allows real-time visualization of immune cell dynamics and interactions in vivo. Moreover, the genetic tractability of zebrafish has greatly facilitated the molecular and cellular study of immune cells. Thanks to advances in transgenic and genome editing strategies, creating transgenic and mutant zebrafish lines has become convenient and affordable for most labs. Till now, a diverse repertoire of reporter lines has been established for various zebrafish immune cells (Martins et al. 2019). Numerous mutant lines of important molecular regulators have also been generated to investigate the mechanisms underlying the development and functions of immune cells (Hu and Jing 2023).

Among all types of immune cells, macrophages are the most extensively studied in zebrafish. They exhibit conserved phenotypical and functional signatures as their mammalian counterparts, expressing a range of macrophage-specific genes and demonstrating robust phagocytic abilities. Multiple reporter lines of zebrafish macrophage have been generated (Ellett et al. 2011; Dee et al. 2016; Walton et al. 2015), and key regulators governing their development and functions have been identified through meticulous forward and reverse genetic studies (Herbomel et al. 2001; Wu et al. 2018; Yu et al. 2017). Similar to mammals, the development of zebrafish macrophages is orchestrated by a series of esteemed macrophage regulators, such as PU.1/SPI-1 (Spi1b and Spi1a in zebrafish), IRF8, MAFB (Mafba and Mafbb in zebrafish), and CSF1R (Csf1ra and Csf1rb in zebrafish) (Wu et al. 2018; Yu et al. 2017; Lou et al. 2022; Li et al. 2011; Shiau 2015; Oosterhof et al. 2018). As a teleost, zebrafish underwent an additional genome duplication during evolution compared to other higher organisms (Pasquier 2016). This gene duplication generates two orthologues for important macrophage regulators like PU.1 and CSF1R, which gives zebrafish an exceptional advantage in studying their regulatory functions in vivo. In mammals, mutations in these critical regulators are often lethal. However, in zebrafish, the sub-functionalization of duplicated genes typically allows the survival of single mutants, enabling the functional study of each orthologue. Moreover, many of these duplicated genes have experienced functional diversification and acquired new functions during evolution, which may provide new regulatory mechanisms underlying the development and functions of zebrafish macrophages. Therefore, by elucidating the intricate regulatory networks that govern macrophage biology in zebrafish, researchers can gain valuable insights into developing innovative strategies for manipulating macrophage generation and function.

Zebrafish are widely recognized for their remarkable regenerative capabilities. Unlike mammals, zebrafish can regenerate complex tissues throughout their lifespan, including the heart, spinal cord, fins, and even parts of the brain (Kizil et al. 2012; Gemberling et al. 2013). This unique regenerative ability has made zebrafish an invaluable model organism for studying tissue repair and regeneration. Numerous regeneration models regarding different organs have been established in zebrafish (Marques 2019). Combined with the powerful imaging advantages and genetic manipulability, it enables in-depth investigations into the cellular and molecular mechanisms underlying successful tissue regeneration. Besides, these regeneration models are widely employed to unravel the molecular mechanisms underlying human diseases associated with impaired repair and regeneration.

Most macrophage and regeneration studies in zebrafish have predominantly focused on embryos and larvae because of their optical transparency and ease of handling. However, the immune system and organs at these early developmental stages are not fully developed, limiting the ability to capture the intricacies of tissue and immune conditions observed in adult organs. In recent years, there has been a growing emphasis on investigating macrophage function and regeneration in adult zebrafish. A series of techniques and tools have also been developed to facilitate such studies. For example, the generation of a mutant zebrafish line lacking pigment cells has enabled imaging of internal organs in adult zebrafish (White et al. 2008). The advancements in imaging techniques, including multiphoton microscopy, have also significantly enhanced the visualization of cells in deep anatomical regions (Chow et al. 2020; Manley et al. 2020). Additionally, the development of the intubation-based anesthesia procedure has made long-term imaging of live adult zebrafish possible (Castranova et al. 2022; Xu et al. 2015). Alongside these technical advancements, extensive studies have examined the biology of macrophages in adult zebrafish organs, encompassing their formation, maintenance, and functions. Diverse regeneration models in adult zebrafish have also been established (Martins et al. 2019). All these endeavors have positioned adult zebrafish as a promising model for studying macrophages and unraveling their diverse roles in repair and regeneration.

Despite the identification of diverse resident tissue macrophage (RTM) subsets with distinct ontogenies, phenotypes, and locations in various organs across different animal models, current understanding of their specific roles in tissue injury and repair is limited. Investigating the precise functions of these RTM subsets in regeneration represents an important avenue for further research. Understanding how they collaborate to exert diverse and appropriate functions at different repair stages is crucial for unraveling the mechanisms underlying successful regeneration and developing precision therapies for related diseases. Besides, systematic characterization and comparison of RTM compositions between different organs and development stages would be valuable, as the composition of RTM subsets may contribute to the varied regenerative capacity observed among different organs and during different stages of organ development.

## Ontogeny and heterogeneity of macrophages

**Fig. 1:**
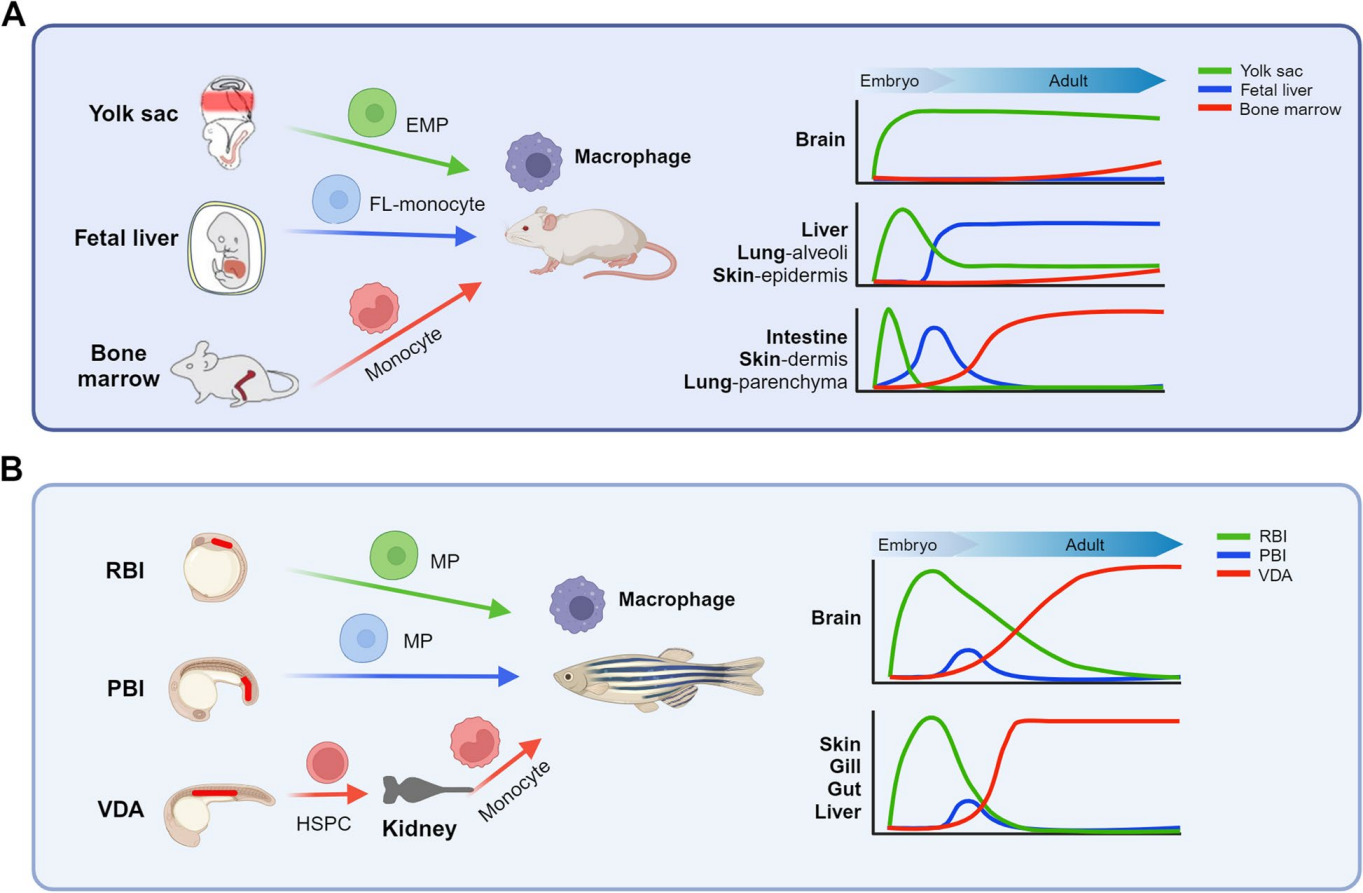
Heterogenous ontogeny of RTMs in mice and zebrafish. **A** RTMs in mice originate from multiple waves of hematopoiesis occurring in distinct hematopoietic tissues during development, including the yolk sac, which produces erythro-myeloid progenitors (EMPs); the fetal liver (FL), which generates a special population of FL-monocytes; and the bone marrow, which gives rise to monocytes. Macrophages from these three waves colonize various tissues and exhibit different replacement kinetic. In the brain, EMP-derived macrophages can self-maintain throughout life and are minimally replaced by macrophages from the other two waves. In the liver, lung alveoli, and epidermis, EMP-derived macrophages are gradually replaced by macrophages derived from FL-monocytes during development, with limited contribution from bone marrow-derived monocytes. However, in the intestine, lung parenchyma, and skin dermis, EMP-derived macrophages are rapidly replaced by macrophages derived from FL monocytes, which are also gradually replaced by macrophages derived from bone marrow monocytes during development. **B** Similar to mice, RTMs in zebrafish also originate from multiple waves of hematopoiesis occurring in distinct hematopoietic site. During early embryonic development, the rostral blood island (RBI) and posterior blood island (PBI) contribute to the production of transient macrophage progenitor cells (MPs), with RBI-derived macrophages being the predominant population. In addition, definitive hematopoiesis takes place in the ventral wall of the dorsal aorta (VDA), generating HSPCs that migrate to the kidney marrow. The kidney marrow serves as a continuous source of monocytes throughout the lifespan. In most zebrafish tissues, RBI and PBI-derived macrophages are rapidly replaced by macrophages derived from monocytes during development, except in the brain where the replacement of RBI-derived macrophages occurs at a slower rate

Under normal conditions, animal organs are populated by a substantial number of RTMs that play crucial roles in tissue development and homeostasis maintenance. Historically, it was believed that these RTMs originated exclusively from monocytes, which are produced by hematopoietic stem cells (HSCs) in the bone marrow (Gordon 2007). However, through a series of seminal lineage-tracing studies in mice over the past decade, it has been revealed that RTMs can arise from both embryonic precursors and circulating monocytes (Fig.1A) (Ginhoux and Guilliams 2016). In particular, during embryonic development, some macrophages originate from alternative hematopoiesis processes that occur in different anatomical locations, such as the yolk sac (YS) and fetal liver (FL), before the establishment of the bone marrow. These early macrophages can persist into adulthood and self-maintain within tissues. As a result, the RTMs in adult organs are composed of a heterogeneous population derived from different origins (Fig.1A). Notably, the proportion of these ontogenically distinct macrophage populations differs between organs. Moreover, the composition of RTMs within a given organ can change over time, influenced by factors such as tissue development, aging, and inflammation (Bleriot et al. 2020). For example, Langerhans cells (LCs), the RTMs in the epidermis, initially generate from the YS-derived progenitors and then are partially replenished by FL-derived progenitors during prenatal development. These prenatal LCs become long-lived cells with little contribution from monocytes in adulthood (Hoeffel et al. 2012). Alveolar macrophages in the lung exhibit a similar dynamic pattern to LCs, with one key distinction that FL-derived alveolar macrophages totally replace YS-derived cells during prenatal development (Guilliams et al. 2013). However, for RTMs in the dermis and lung parenchyma, embryonic macrophages are gradually replaced by monocyte-derived macrophages during postnatal development, and all macrophages in these tissues are derived from monocytes in adulthood (Tamoutounour et al. 2013; Chakarov et al. 2019). Similarly, embryonic RTMs in the intestine are quickly replaced by monocyte-derived macrophages during development (Bain et al. 2014).

Zebrafish studies have also made significant contributions to our understanding of the origin and ontogeny of RTMs. Like mammals, zebrafish undergo multiple waves of hematopoiesis at different anatomical locations during development (Fig. 1B). In the early embryonic stage, two major waves of hematopoiesis take place. The first wave, primitive hematopoiesis, occurs in the intermediate cell mass and rostral blood island, giving rise to red blood cells and myeloid cells. The second wave, called definitive hematopoiesis, initiates at the ventral wall of the dorsal aorta and produces HSPCs. These HSPCs then migrate to other hematopoietic tissues and ultimately colonize the kidney marrow, which is the zebrafish equivalent of the mammalian bone marrow, supporting larval and adult hematopoiesis (Stachura et al. 2016). The external development of zebrafish embryos offers a unique opportunity to study the hematopoietic origins of RTMs with high spatial and temporal resolution. To comprehensively investigate the origins and dynamics of zebrafish RTMs, our group has developed an innovative infrared laser-induced in vivo cell labeling and lineage tracing system. Through this system, we have discovered that similar to that in mice, zebrafish RTMs can arise from both primitive and definitive hematopoiesis. As development progresses, the primitive RTMs are gradually replaced by definitive RTMs (Xu et al. 2015). However, while some adult mouse organs, such as the brain, skin, and liver, retain a population of RTMs derived from primitive hematopoiesis, our study has demonstrated that RTMs in these adult zebrafish organs exclusively originate from HSPCs of definitive hematopoiesis (Fig.1B) (He 2018). This finding has also been supported by other conventional lineage tracing studies using the promoter-controlled CreER-loxP system (Ferrero et al. 2018; Ferrero 2020). While this discrepancy between zebrafish and mouse RTMs may be attributed to species differences, it is also worth considering the potential influence of different housing conditions on RTM dynamics in these two model organisms. Most RTM development studies in mice are conducted on animals housed in specific pathogen-free conditions, which minimize exposure to environmental microbes. On the other hand, zebrafish are typically housed in near-wild conditions and exposed to a more diverse array of microorganisms. It is worth noting that environmental factors, especially microbes, can regulate the survival and maintenance of RTMs (Bleriot et al. 2020).

In addition to their heterogeneous ontogenies, RTMs in adult organs were also found to consist of various subsets with distinct phenotypical features and sub-tissue locations. For example, while the intestinal RTMs in adult mice are exclusively derived from monocytes, they can be further classified into phenotypically and functionally distinct subsets based on their specific locations or turnover rates (Shaw et al. 2018; Mowat et al. 2017). Recent advancements in single-cell omics studies have provided unprecedented insights into the compositional heterogeneity of RTMs within diverse organs across different organisms (Li et al. 2022; Domanska 2022; Guilliams et al. 2022; Zilionis et al. 2019). Notably, these studies have revealed some functionally or distributionally conserved RTM subsets across multiple organs (Chakarov et al. 2019; Dick et al. 2022). Among these subsets, two of the most well-established ones are the nerved-associated Lyve1^lo^MHCII^hi^ macrophages and vessel-associated Lyve1^hi^MHCII^lo^macrophages. The specific association of these macrophages with nerves or blood vessels endows them with unique functions closely related to these anatomical structures. Additionally, these macrophages exhibit committed phenotypes and limited plasticity (Chakarov et al. 2019; Ural 2020). These findings suggest that functionally committed RTM subsets coexist within various organs, collectively contributing to the comprehensive functional repertoire of macrophages. Our recent study has also identified these kinds of committed macrophage subsets in zebrafish (Zhou et al. 2023). Through scRNA-seq analysis of RTMs in multiple adult zebrafish organs, we have discovered that RTMs in most organs can be primarily classified into two molecularly conserved subsets: the pro-inflammatory macrophages with potent phagocytosis and pro-inflammatory signatures, and pro-remodeling macrophages with tissue remodeling and pro-regeneration signatures (Zhou et al. 2023). The unique transcriptional profiles of these two macrophage subsets, particularly their associations with inflammation and tissue remodeling, strongly imply their different roles in tissue repair and regeneration processes, which warrants further investigation.

## Functions of macrophages during tissue repair and regeneration

**Fig. 2:**
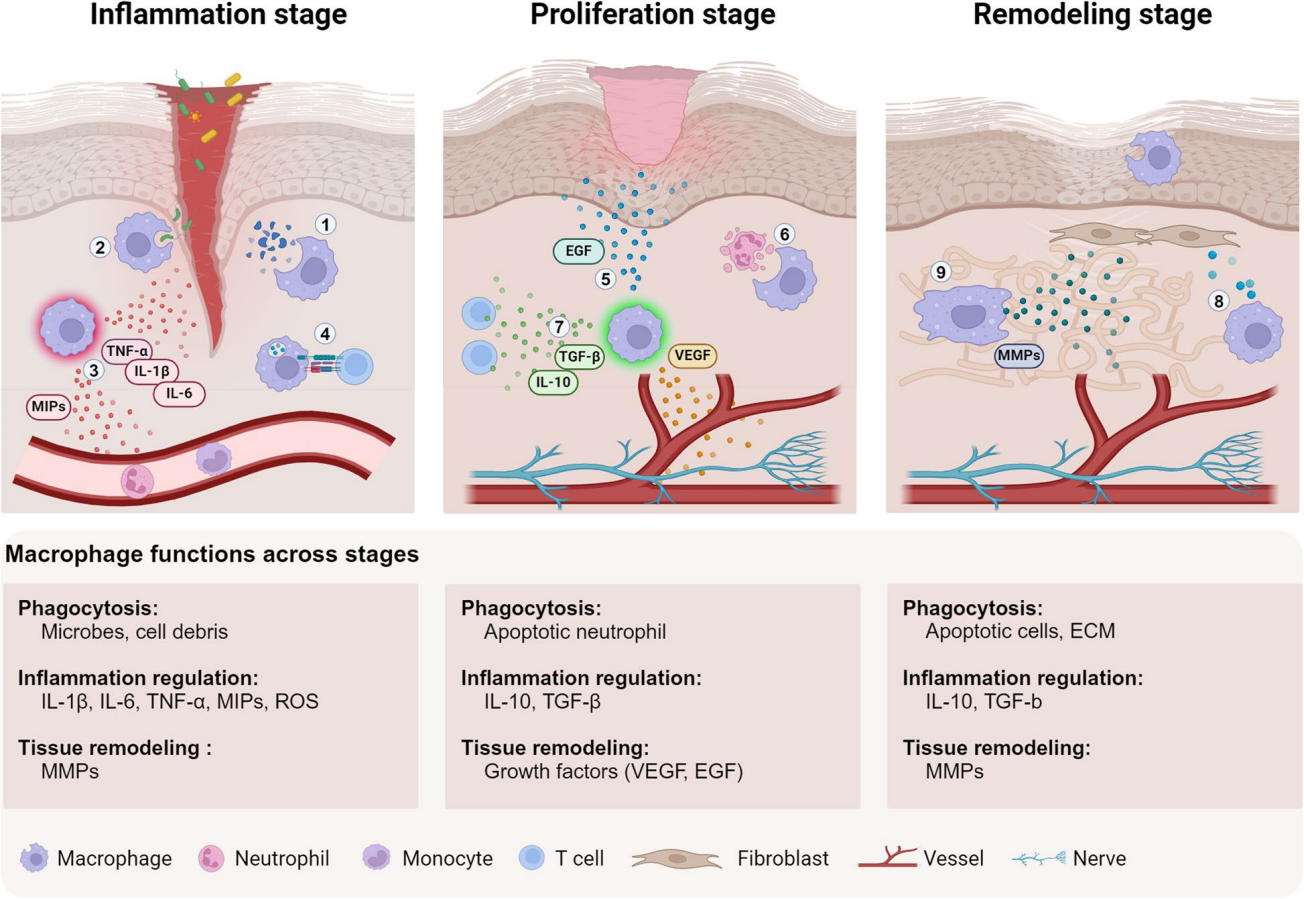
Diverse functions of macrophages during tissue regeneration. This illustration delineates the three main stages of skin wound healing and the key functions of macrophages across these stages. Macrophages perform three primary types of functions: phagocytosis, inflammation regulation, and tissue remodeling. At the inflammation stage, macrophages are responsible for the removal of cell debris (①) and microbes (②) through phagocytosis. Besides, they can stimulate tissue inflammation by releasing pro-inflammatory cytokines and chemokines (③), which leads to the recruitment and activation of other resident immune cells, as well as circulating monocytes and neutrophils. Additionally, macrophages can degrade the ECM to facilitate the infiltration of these recruited cells. Under certain conditions, macrophages can also activate tissue-resident T cells to leverage adaptive immunity for inflammation regulation (④). At the proliferation stage, macrophages promote the differentiation and proliferation of various tissue cells, including epidermal cells, stromal cells, and endothelial cells, through the production of growth factors (⑤). Besides, macrophages can clear apoptotic neutrophils (⑥) and produce anti-inflammatory modulators to resolve tissue inflammation (⑦). At the remodeling stage, macrophages promote the synthesis and deposition of ECM components by regulating the activities of ECM-producing cells such as fibroblast (⑧). They also regulate the formation of ECM structure by releasing ECM-modifying enzymes like MMPs (⑨). Besides, macrophages can guide the formation of neural and vascular networks within the tissues

Despite variations in the injury and tissue type, the tissue injury response and repair process exhibit fundamental similarities. It can typically be divided into three main stages: the inflammatory stage, the proliferation stage, and the remodeling stage (Fig. 2). Upon injury, the body immediately initiates an inflammatory response to control bleeding, eliminate pathogens, and remove damaged tissue. The proliferation stage involves the renewal and rebuilding of damaged tissues by the proliferation of tissue cells. The final stage is remodeling, where the newly formed tissue undergoes maturation and remodeling to restore its architecture and functionality (Gurtner et al. 2008).

Macrophages are multifaceted cells that perform diverse functions at different stages of the repair process (Fig. 2). In addition to their prominent role in scavenging dead cells and eliminating invading pathogens at the injury site, macrophages also play a central role in modulating the inflammatory state by secreting various inflammation mediators. Moreover, they actively participate in tissue remodeling by modifying the extracellular matrix (ECM). Through the secretion of various trophic factors, macrophages also promote the proliferation and maturation of different tissue cells, thereby facilitating tissue architecture and functionality reconstitution. These diverse functions of macrophages are vital for successful regeneration, as perturbations in these activities can lead to aberrant repair outcomes, such as chronic wounds and fibrosis (Wynn and Vannella 2016). As zebrafish have been widely utilized for macrophage and regeneration study, the versatile roles of macrophages in repair and regeneration have also been demonstrated in this model organism. Remarkably, the dynamic behavior and activities of macrophages during regeneration are elegantly visualized through exquisite intravital imaging studies (Bohaud et al. 2021).

### Phagocytosis

Tissue injury often results in extensive cell death and possibly the invasion of pathogens when it happens to barrier tissues. Timely removal of these deceased cells and invading pathogens is crucial to initiate tissue repair promptly, and failure in their clearance can lead to prolonged cell death and delayed repair. Macrophages are the principal phagocytes responsible for this important task. They are equipped with a wide range of receptors that enable them to recognize and engulf different types of particles. These receptors encompass Fc receptors, complement receptors, and scavenger receptors, which are responsible for engulfing deceased cells and debris (Uribe-Querol 2020). Additionally, macrophages express C-type lectin receptors, a family of membrane-bound pattern recognition receptors (PRRs), to mediate the phagocytosis of pathogens (Li and Underhill 2020). Toll-like receptors, another type of membrane-bound PRR expressed by macrophages, do not directly initiate phagocytosis but enhance the activity of other phagocytic receptors (Fu and Harrison 2021). Notably, these phagocytic receptors on the macrophage membrane have served as hallmarks for identifying macrophages across different mammalian organs (Cox et al. 2021).

Neutrophils are another important phagocytic cell population in repair and infection. However, unlike macrophages with a broader phagocytic repertoire, neutrophils are highly specialized and remarkably efficient in engulfing and destroying invading pathogens, including bacteria and fungi (Silva 2010). As the first responders to sites of injury, the effective elimination of pathogens by neutrophils is critical for preventing exacerbated tissue damage resulting from infection. Comprehensive characterization and comparison of the phagocytic abilities of macrophages and neutrophils during tissue repair have been conducted in embryonic zebrafish (Keightley et al. 2014). These studies unveiled that neutrophils are the primary scavengers at the inflammatory stage, while macrophages become dominant phagocytes at the resolution stage (Li et al. 2012).

Besides clearing cell debris and eliminating pathogens, the phagocytosis process also regulates other functions and activities of macrophages. It can reshape the transcriptional and metabolic program of macrophages and regulate their migration and proliferation (Eming et al. 2017; Gerlach et al. 2021). Notably, these regulatory roles can vary depending on the immunological microenvironment and the phagocytosed objects. For example, during the early inflammation stage of the injury response, when substantial harmful agents are present, macrophage phagocytosis leads to the production of significant amounts of reactive oxygen species (ROS). These pro-inflammatory agents can further enhance the phagocytic activity of macrophages and attract other phagocytes to the site of injury, contributing to the efficient clearance of pathogens and cellular debris (Mittal et al. 2014). During the later stage of the repair process, as inflammation subsides, a considerable number of neutrophils undergo apoptosis after fulfilling their roles, and macrophages are responsible for their clearance. However, unlike in the early inflammation stage, the phagocytosis of apoptotic neutrophils does not further enhance inflammation. Instead, it promotes the proliferation of macrophages and triggers a pro-regeneration program within them (Gerlach et al. 2021; Bouchery and Harris 2019). Importantly, this program shift is essential for resolving inflammation and initiating the repair process. Overall, these mechanisms underscore the profound significance and broad relevance of macrophage phagocytosis in regulating the orderly progression of the repair process.

### Inflammation regulation

The inflammatory response following tissue injury and its subsequent resolution during wound healing are crucial processes in tissue repair. They are tightly regulated by a complex interplay of cellular and molecular effectors. Dysregulation of these processes can have detrimental consequences, including chronic wounds or fibrotic repair (Eming et al. 2017). Macrophages act as key orchestrators in this regulatory framework. They continuously sense and respond to the evolving needs of the repair process, thereby regulating the duration and intensity of inflammation. Macrophages are equipped with a diverse repertoire of receptors to integrate signals from the surrounding milieu, and they can modulate the behavior and activity of neighboring cells through direct interactions and the production of various modulators, including cytokines, chemokines, complement components, and metabolites (Watanabe et al. 2019).

During the early stage of inflammation, macrophages respond to signals from damaged tissues and invading pathogens by initiating a pro-inflammatory program. These activated macrophages rapidly release various pro-inflammatory mediators, including well-known cytokines such as TNFα, IL-1β, and IL-6. These cytokines serve multiple functions, including promoting the apoptosis of damaged or infected cells to aid in their clearance, amplifying the inflammatory response by inducing the production of more pro-inflammatory mediators, and stimulating the activation of other immune cells at the site of injury (Krzyszczyk et al. 2018). Macrophages also secrete a specific family of chemokines called macrophage inflammatory proteins (MIPs) to guide the directed migration of circulating inflammatory cells toward the site of inflammation (Maurer and von Stebut 2004). Besides, macrophages produce matrix metalloproteinases (MMP-2 and MMP-9) to break down the ECM in the injury site, making room for these infiltrated cells and facilitating their movement. In addition to their secretion of pro-inflammatory mediators, pro-inflammatory macrophages can activate tissue-resident T cells through antigen presentation (Krzyszczyk et al. 2018). This interaction enables the involvement of adaptive immunity in the inflammatory response, further enhancing the immune response. While at the tissue repair stage, macrophages adopt a pro-regeneration program. These pro-resolving macrophages suppress the inflammation response by producing anti-inflammatory cytokines such as IL-10 and TGF-β (Watanabe et al. 2019).

Besides protein regulators, small molecule metabolites produced by macrophages can also act as inflammatory mediators. Recent studies have revealed that macrophages adopt different metabolic strategies at various stages of tissue repair, and the produced metabolites can influence the transcriptional program of macrophages and modulate the activity of neighboring cells (Eming et al. 2017; Bohaud et al. 2022; Paredes 2021). Typically, pro-inflammatory macrophages rely on glycolysis as their primary energy production pathway to adapt to the hypoxic microenvironment of the injury site and meet the acute energy demand of activated cells. This metabolic pathway leads to the production of various pro-inflammatory mediators, such as nitric oxide and ROS. On the other hand, pro-resolving macrophages preferentially utilize oxidative phosphorylation, which can meet the high energy demands of the pro-regeneration program without generating pro-inflammatory metabolites. Despite extensive studies on the regulatory roles of cell metabolism in inflammation modulation, the underlying mechanisms remain largely unexplored. To further investigate and understand these mechanisms, advanced techniques and tools are needed to trace and examine the activities of these metabolites.

Zebrafish studies have contributed significantly to understanding macrophage functions in inflammatory responses. The generation of reporter lines for key inflammatory mediators, such as TNFα and IL-1β, has allowed for the dynamic tracing of the inflammatory response and visualization of the behavior of pro-inflammatory macrophages. By utilizing these lines, Tsarouchas et al. discovered that TNFα and IL-1β play different roles in inflammation regulation, and the dynamic control of their expression by peripheral macrophages is vitally important for the spinal cord regeneration in zebrafish (Tsarouchas et al. 2018). Additionally, in the context of zebrafish heart regeneration, it was observed that*tnfa* + macrophages promote scar deposition, while *tnfa*- macrophages facilitate scar removal (Bevan et al. 2020). Moreover, by utilizing specific probes of various metabolites or detecting their inherent autofluorescence, researchers have traced the intracellular metabolism of macrophages during tissue damage and repair (Paredes et al. 2018; Miskolci 2022). More recently, through single-cell RNA sequencing (scRNA-seq) analysis, Tatjana Piotrowski’s group discovered that macrophages undergo three sequential states of anti-inflammatory activation during hair cell regeneration. Although each state is independently activated, they work synergistically to promote regeneration (Denans et al. 2022).

Despite the extensive studies conducted thus far, there are still important unanswered questions regarding the roles of macrophages in inflammation regulation. One crucial question is how macrophages integrate diverse signals from the tissue microenvironment to determine their specific regulatory program. Another key question is whether pro-inflammatory and anti-inflammatory macrophages represent different activation states of the same cells or whether they are ontogenically distinct populations. With numerous genetic tools available in zebrafish to mark and analyze these cells, studies in zebrafish could significantly contribute to answering these questions.

### Tissue remodeling

At the later stage of tissue repair, various tissue cells undergo proliferation and reorganization to regenerate different structures, including epithelial barriers, blood and lymphatic vessels, nerves, and other functional architectures. Macrophages play multiple crucial roles in these processes. Firstly, they promote the activation of tissue stem/progenitor cells, driving their differentiation and proliferation. Besides, macrophages regulate the distribution of newly generated cells, ensuring their proper migration and integration into the damaged tissue. Macrophages also contribute to the remodeling of the ECM, which is vital for tissue restructuring and functional restoration (Wynn and Vannella 2016).

Zebrafish studies have unveiled many critical and conserved mechanisms underlying the pro-remodeling functions of macrophages and provided valuable insights into developing innovative therapeutic interventions for promoting tissue repair in various clinical settings (Bohaud et al. 2021). In a recent study on muscle regeneration in zebrafish, it has been observed that macrophages can form a transient activation niche for muscle stem cells by surrounding them and producing a proliferative signal called NAMPT. Notably, the study demonstrated that the administration of exogenous NAMPT in mouse muscle injuries can stimulate muscle regeneration, highlighting the therapeutic potential of this molecule (Ratnayake et al. 2021). Furthermore, zebrafish studies have revealed that the pro-inflammatory signal TNF can also stimulate the proliferation of progenitor cells during skin and spinal cord injuries (Cavone et al. 2021; Nguyen-Chi 2017). It suggests that cell proliferation is not exclusive to the inflammation-resolving stage but occurs much earlier. In addition to secreting trophic factors by themselves, macrophages were also found to stimulate other tissue cells to produce these proliferative signals, thereby accelerating the remolding process (Bruton et al. 2022). Interestingly, Liu et al. discovered that macrophages could also mediate the repair of blood vessels by directly adhering to separated endothelial cells and pulling them together (Liu et al. 2016).

ECM remodeling during tissue repair involves the breakdown of damaged ECM components and the synthesis and deposition of new ECM elements. Macrophages contribute to ECM remodeling through various mechanisms. Firstly, they secrete MMPs to break down collagen, elastin, and other ECM components, facilitating the clearance of damaged matrix materials. This process is essential for creating space for new tissue growth and remodeling. Macrophages also participate in ECM synthesis and deposition. They can produce and release factors such as growth factors, cytokines, and chemokines that promote the migration and proliferation of fibroblasts, which are the primary cells responsible for ECM synthesis. Macrophages can also directly interact with fibroblasts and other ECM-producing cells, providing them with signals and cues to enhance ECM production. Furthermore, other pro-remodeling functions of macrophages can also influence the ECM remodeling process. For example, macrophages promote angiogenesis, the formation of new blood vessels, which is essential for delivering oxygen and nutrients to regenerating tissues and facilitating ECM remodeling (Sutherland et al. 2023).

Notably, the pro-remodeling functions of macrophages are also crucial for supporting normal tissue development. For example, macrophages were discovered during embryonic development to promote the movement and colonization of hematopoietic stem and progenitor cells (HSPCs) by remodeling ECM along their migration pathway (Travnickova 2015). Additionally, macrophages in the larval fish skin have been found to play a crucial role in relaying signals between pigment cells, thus regulating the formation of the pigment pattern (Eom and Parichy 2017).

### Functional plasticity of macrophages

Macrophages are known as highly plastic cells. They can dynamically alter the morphologies and behaviors in response to different microenvironmental cues, thus allowing them to exert a broad spectrum of functions in health and diseases (Locati et al. 2020). The M1/M2 theory has long been a paradigm in interpreting macrophage plasticity. It describes M1 and M2 macrophages as two distinct activation states with different phenotypes and functions. M1 macrophages, or classically activated macrophages, are stimulated by signals like interferon-gamma (IFN-γ) and microbial products. They play a critical role in pathogen defense by performing phagocytosis, producing inflammatory cytokines, and generating ROS. In contrast, M2 macrophages, or alternatively activated macrophages, are stimulated by IL-4, IL-13, and IL-10 signals. They are associated with anti-inflammatory and tissue repair functions (Martinez 2014). In tissue repair and regeneration, it is believed that the pro-inflammatory functions of M1 macrophages are important for the early stages of injury response, while M2 macrophages are involved in dampening inflammation and promoting tissue remodeling and regeneration (Kim and Nair 2019). While M1 and M2-like macrophages are well identified and described during tissue injury and repair, it has been challenging to determine whether they represent different polarized activation states of the same macrophage population or ontogenically distinct cell populations. Notably, through the in vivo fate tracing of*tnfa*+ pro-inflammatory macrophages in a zebrafish injury model, Nguyen-Chi et al. demonstrated that polarized M1 macrophages can indeed undergo a phenotypic switch and convert into M2-like phenotypes during the resolution stage of tissue repair (Nguyen-Chi 2015). This finding has provided compelling evidence for the contribution of macrophage plasticity to their diverse functions during tissue repair and regeneration.

However, the M1/M2 paradigm has been increasingly recognized as an oversimplified concept. Over the past two decades, extensive research has revealed that macrophages possess a much broader functional repertoire than initially thought. The dichotomy of M1 and M2 macrophages represents only two extremes along a spectrum of macrophage activation states (Martinez 2014). In fact, with the identification of additional phenotypical markers, the category of M2 macrophages has been further subdivided into subtypes such as M2a, M2b, M2c, and M2d (Roszer 2015). The recent advent of single-cell and genomic technologies has added even more complexity to our understanding of this process and revealed a high degree of heterogeneity and dynamic nature in macrophage activation (Sanin et al. 2022). Nowadays, a continuous spectrum model of macrophage activation has been more widely accepted. Instead of categorizing macrophages into discrete activation states based on a limited number of phenotypical markers, this model takes into account a combination of macrophage features, including their gene expression profiles, metabolic characteristics, and interactions with the microenvironment (Sanin et al. 2022; Guilliams 2015). This comprehensive approach better captures the multifaceted nature of macrophages and allows for a more nuanced understanding of their functions. Recent scRNA-seq studies in zebrafish have also supported this notion by revealing the continuous and dynamic activation process of macrophages during heart and neuromast regeneration (Denans et al. 2022; Hou et al. 2020; Wei 2023).

Despite this significant progress in understanding macrophage plasticity, important questions remain to be answered. One key question is whether macrophages of different tissues or origins display plasticity differences. Investigating whether macrophages from distinct sources have inherent variations in their ability to assume the full spectrum of activation states is intriguing. Another critical question is whether macrophages in different activation states can freely transition back to a resting state or shift into alternative activation states. Elucidating the dynamics of macrophage plasticity, including the reversibility and flexibility of their activation states, is crucial for comprehending their functional adaptability and potential therapeutic implications. Lastly, the mechanisms underlying the functional plasticity of macrophages and the key factors determining their state-shifting need further investigation. It may involve a complex interplay between various factors, including signaling pathways, transcriptional regulation, epigenetic modifications, and interactions with the microenvironment. Comprehensive studies utilizing advanced techniques such as multi-omics approaches, intravital live-cell imaging, and lineaging tracing are required to address these questions. With its unique advantages in employing these techniques, zebrafish thus holds great promise as an ideal model organism for conducting such studies.

## RTMs VS monocyte-derived macrophages during tissue repair and regeneration

Upon tissue injury, circulating monocytes would be recruited to the injury site and differentiate into effector macrophages. These monocyte-derived macrophages (MoMs) actively participate in tissue repair and exhibit distinct and overlapping roles compared to RTMs (Wynn and Vannella 2016). Exploring the functional and developmental relationship between MoMs and RTMs in tissue repair has been a long-standing research focus. Various strategies are available in mice to distinguish these two types of macrophages, including distinct phenotypic markers, adoptive cell transfer, and genetic lineage tracing. In most studies, MoMs are revealed as a transient population of activated macrophages that primarily play pro-inflammatory and immune defense roles during the early stages of injury response. In contrast, RTMs exhibit an anti-inflammatory and tissue-protective profile throughout the injury response and repair process (Schneider et al. 2014). At the early stage of inflammation, the number of MoMs often greatly exceeds the population of RTMs. However, most MoMs undergo apoptosis and are cleared by RTMs after inflammation resolution. The opposite and complementary activities of MoMs and RTMs are believed to be crucial for regulating tissue inflammation and coordinating the repair process (Roquilly et al. 2022).

However, with the identification of diverse RTM subsets, it was found that certain RTM subsets also have pro-inflammatory functions (Asano et al. 2015). Meanwhile, through the analysis of MoMs at a higher resolution, it has been realized that their roles in tissue repair are much more complex than previously thought. Recent studies have identified the existence of both anti-inflammatory and pro-regenerative MoMs during the repair process (Sajti et al. 2020). Moreover, after tissue restoration, certain MoMs were observed to acquire the phenotypic characteristics of RTMs and occupy the vacant spaces left by the deceased RTMs during tissue injury (Guilliams and Scott 2022). These findings demonstrated the significant plasticity of MoMs and highlighted their ability to perform functions similar to RTMs. Despite that, the comprehensive understanding of the functional and developmental relationships between MoMs and RTM subsets is still limited and requires further investigation.

Current understanding of MoMs in zebrafish and their contribution to tissue regeneration is limited because most zebrafish regeneration studies have focused on the embryonic and larval stages, during which MoMs are not yet present. Recently, a growing interest has been in developing regeneration models using adult zebrafish due to their possession of more complex tissue structures and immune cell composition, which better resemble the condition in mammalian organs (Marques 2019; Wattrus and Zon 2021). Through bulk and single-cell RNA sequencing analysis of macrophages involved in adult tissue regeneration, the heterogeneous composition of macrophages was observed, and specific subsets of macrophages with pro-inflammatory and pro-regenerative functions were identified (Wei 2023; Sanz-Morejon et al. 2019). However, whether these functional macrophage subsets belong to RTMs or are derived from monocytes is unknown. Deciphering this issue needs the specific labeling and lineage tracing of monocytes and distinct RTM subsets during tissue regeneration. Notably, the recent generation of specific reporter lines for these macrophage populations and the development of advanced cell lineage tracing strategies have paved the way for pursuing these comprehensive studies in zebrafish (Zhou et al. 2023; He 2020).

## Conclusions

Currently, it has become evident that both the heterogeneous composition and functional plasticity of macrophages contribute to their multifaceted roles in tissue repair and regeneration. Therefore, to decipher the complicated regulatory network of macrophages in tissue repair, it is essential to identify the bona fide macrophage subsets within the tissue and track their dynamic responses and functions during the repair process by lineage tracing. Investigating the molecular mechanisms underlying macrophage subset differences and functional plasticity is also intriguing. This knowledge will deepen our understanding of macrophage biology and aid in discovering therapeutic targets for related diseases. With continuous advancements in tools and techniques, zebrafish research offers great potential for advancing these studies and expanding our knowledge of macrophage functions in tissue repair and regeneration.

## Data Availability

Not applicable.

## Abbreviations

ECM: Extracellular matrix
FL: Fetal liver
HSPC: Hematopoietic stem and progenitor cell
HSC: Hematopoietic stem cell
IFN-γ: Interferon-gamma
LC: Langerhans cell
MIP: Macrophage inflammatory protein
MMP: Matrix metalloproteinases
MoM: Monocyte-derived macrophage
PRR: Pattern recognition receptor
ROS: Reactive oxygen species
RTM: Tissue-resident macrophage
scRNA-seq: Single-cell RNA sequencing
YS: Yolk sac
